# Optimal Coherence Length Control in Interferometric Fiber Optic Hydrophones via PRBS Modulation: Theory and Experiment

**DOI:** 10.3390/s25154711

**Published:** 2025-07-30

**Authors:** Wujie Wang, Qihao Hu, Lina Ma, Fan Shang, Hongze Leng, Junqiang Song

**Affiliations:** College of Meteorology and Oceanology, National University of Defense Technology, Changsha 410073, China; wangwujie@nudt.edu.cn (W.W.); huqihao18@nudt.edu.cn (Q.H.); mln_c7@nudt.edu.cn (L.M.); shangfan18@hotmail.com (F.S.); hzleng@nudt.edu.cn (H.L.)

**Keywords:** interferometric fiber optic hydrophone, coherence length, parasitic interference noise, pseudo-random binary sequence

## Abstract

Interferometric fiber optic hydrophones (IFOHs) are highly sensitive for underwater acoustic detection but face challenges owing to the trade-off between laser monochromaticity and coherence length. In this study, we propose a pseudo-random binary sequence (PRBS) phase modulation method for laser coherence length control, establishing the first theoretical model that quantitatively links PRBS parameter to coherence length, elucidating the mechanism underlying its suppression of parasitic interference noise. Furthermore, our research findings demonstrate that while reducing the laser coherence length effectively mitigates parasitic interference noise in IFOHs, this reduction also leads to elevated background noise caused by diminished interference visibility. Consequently, the modulation of coherence length requires a balanced optimization approach that not only suppresses parasitic noise but also minimizes visibility-introduced background noise, thereby determining the system-specific optimal coherence length. Through theoretical modeling and experimental validation, we determined that for IFOH systems with a 500 ns delay, the optimal coherence lengths for link fibers of 3.3 km and 10 km are 0.93 m and 0.78 m, respectively. At the optimal coherence length, the background noise level in the 3.3 km system reaches −84.5 dB (re: rad/√Hz @1 kHz), representing an additional noise suppression of 4.5 dB beyond the original suppression. This study provides a comprehensive theoretical and experimental solution to the long-standing contradiction between high laser monochromaticity, stability and appropriate coherence length, establishing a coherence modulation noise suppression framework for hydrophones, gyroscopes, distributed acoustic sensing (DAS), and other fields.

## 1. Introduction

Interferometric fiber optic hydrophones (IFOHs), with their high sensitivity, high integration, simple optical structure, potential durability, and reliability [1,2,3,4], are regarded as an important solution for next-generation exploration technologies, such as compact small-diameter towed arrays, laterally large-aperture acoustic arrays [5,6,7,8,9,10,11,12], and permanent seismic monitoring systems on the seafloor [13,14,15,16]. Additionally, distributed acoustic sensing (DAS) based on fiber optic interferometric sensing is conducive to capturing weak signals from the distant seafloor [1,3,17,18,19,20].

IFOHs typically employ highly coherent, narrow-linewidth lasers [21,22,23,24] to achieve large-scale array multiplexing [25,26,27] and improve sensing resolution [28]. However, this use of high-coherence lasers introduces various forms of stray light, such as Rayleigh scattering (RS) [5,24,29], grating reflections [30,31,32], and fiber fusion point-induced backward scattering, which can lead to high-intensity parasitic interference with interference pulses. This interference significantly degrades the accuracy of the sensing signal and increases background noise levels. While phase-generating carrier (PGC) phase modulation can suppress RS noise by approximately 10 dB [33,34], it becomes insufficient for link fiber systems exceeding 10 km, where RS increases significantly. Indeed, adding optical isolators at the light source output, link fiber, and underwater sensor array can effectively suppress parasitic interference, but it increases system complexity [35].

Recently, techniques for controlling laser coherence length have been applied to suppress random walk noise and backscattering noise in fiber optic gyroscopes (FOGs) [36,37,38,39,40,41], primarily utilizing triangular wave [36], dual-tone sinusoidal wave [37] and pseudo-random binary sequence (PRBS) modulation [38,39,40,41]. However, unlike triangular and dual-tone sinusoidal wave schemes, which can introduce spectral instability [36,37], PRBS modulation achieves precise control of laser coherence length without compromising wavelength stability, thereby optimizing noise suppression [39,40].

Owing to the superior performance of PRBS modulation in coherence length regulation, it has recently been adopted in interferometers to mitigate RS noise [5,42,43,44], demonstrating excellent efficacy. Furthermore, our previous research revealed that PRBS modulation exhibits significantly better performance than sinusoidal modulation in IFOH applications [5]. Despite these findings, a critical gap persists in existing studies: the underlying physical mechanism through PRBS modulation suppressing parasitic interference remains unexplained. Therefore, in the present work, we elucidate the mechanism of PRBS phase modulation on background noise in IFOHs, establishing a quantitative relationship between PRBS parameter and laser coherence length. Our findings reveal a direct proportionality between laser coherence length and PRBS bit period. Moreover, the interference visibility is approximately proportional to the laser coherence length, with a coefficient of 0.47. When visibility falls below the IFOH system’s threshold, halving the coherence length induces a 6 dB elevation in background noise due to insufficient visibility. Therefore, although reducing the coherence length can effectively suppress parasitic interference noise, the contradiction of the background noise increase caused by the reduction in interference visibility also needs to be considered, and then the optimal coherence length for the system should be selected. In an IFOH system featuring a 3.3 km link fiber and a matched interferometer with a 500 ns arm difference, the optimal coherence length is determined to be 0.93 m, at which point the background noise level is −84.5 dB, indicating an additional noise suppression of 4.5 dB on top of the original PRBS suppression by determining the optimal coherence length [5].

The theoretical framework and experimental results provide a solution to the long-standing contradiction between high laser monochromaticity, stability and appropriate coherence length, thus reducing the sensor noise, which is applicable to hydrophones, FOGs, DAS, and other related fields.

## 2. Theory and Methodology

### 2.1. Impact of PRBS Phase Modulation on Laser Coherence Length

**Figure 1:**
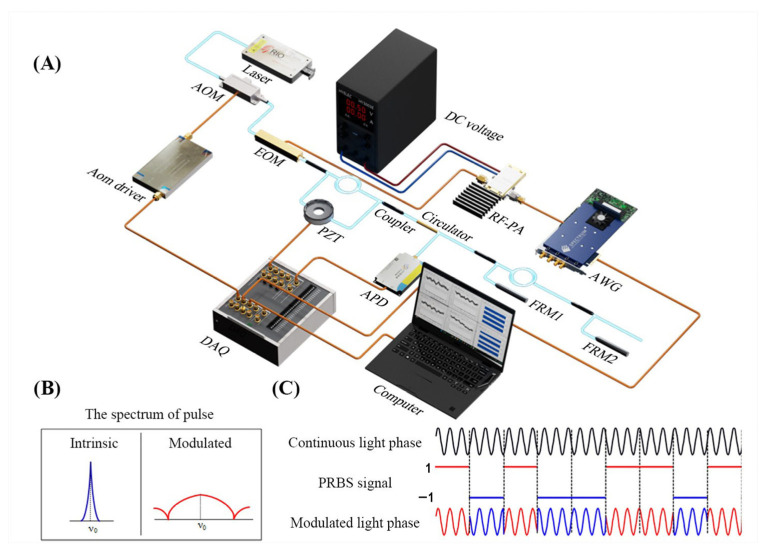
Phase modulation using PRBS. (**A**) Optical path structure diagram of the sensing system with PRBS phase modulation added. (**B**) Spectrum before and after PRBS modulation. (**C**) Schematic illustration of direct phase modulation of the pulsed light field controlled by PRBS.

The IFOH systems, which incorporate PGC modulation and PRBS modulation, are depicted in Figure 1A. Before undergoing interferometric propagation, the optical pulse is phase-modulated by an electro-optic modulator (EOM) driven by a PRBS signal, thereby realizing a controlled pulse modulation scheme. At this stage, the pulsed light can be mathematically expressed as:

(1) $$E_{out}=\exp\left(i2\pi \nu_{0}t+i\phi_{\mathrm{las}}+i\phi_{PRBS}\left(t\right)\right)$$

where $\nu_{0}$ represents the central light frequency, $\phi_{las}$ denotes the phase noise induced by the inherent bandwidth of the light source $\Delta \nu_{0}$, and $\phi_{PRBS}$ refers to the phase modulation caused by the EOM. PRBS exhibits characteristic balanced distributions with statistically equivalent probabilities for binary states (0/1). Hence, a PRBS of high order can be viewed as a random sequence. The magnitude of the phase shift induced by a bit is denoted as $\pm \phi_{0}$. Then, the phase shift induced by PRBS can be expressed as [41]:

(2) $$\phi_{PRBS}\left(t\right)=\frac{\phi_{0}}{2}\sum_{n=-\infty }^{\infty }a_{n}p\left(t-nT\right)$$

where $p\left(t\right)$ represents the pulse shape of PRBS, T is the bit period, $\phi_{0}$ is the peak-to-trough difference, and $a_{n}$ is a random value that takes values ±1 with equal probability. We conducted an analysis of the square-wave pulses:

(3) $$p\left(t\right)=\left\{\begin{array}{cc} 1 & 0<t<T\\ 0 & otherwise \end{array}\right.$$

In accordance with the Wiener–Khinchin theorem, the power spectral density (PSD) of a wide-sense stationary random process is the Fourier transform of its autocorrelation function. Based on Equation (3), the PSD induced by phase modulation $S_{PRBS}\left(\nu \right)$ can be obtained [41]:

(4) $$\begin{array}{cc} S_{PRBS}\left(\nu \right) & =\int_{-\infty }^{\infty }\phi_{PRS}\left(t\right)e^{-j\nu t}dt\\ & =\int_{-\infty }^{\infty }\frac{\phi_{0}}{2}\sum_{n=-\infty }^{\infty }a_{n}p\left(t-nT\right)e^{-j\nu t}dt\\ & =\frac{1}{2}\left(1+\cos\phi_{0}\right)\delta \left(\nu \right)+\frac{T}{2}\left(1-\cos\phi_{0}\right)\sin c^{2}\left(\pi T\nu \right) \end{array}$$

Based on Equation (4), it can be found that, when PRBS is used to phase-modulate the inquiry pulse, it comprises two components: an unmodulated carrier term and a wide spectrum arising from the phase modulation. The relative magnitudes of these two terms are governed by the modulation amplitude $\phi_{0}$. Notably, when $\phi_{0}=\pi$, the unmodulated carrier term diminished to zero, resulting in the entire light source power being distributed across the wide spectral components. Consequently, Equation (4) can be reformulated as:

(5) $$S_{PRBS}\left(\nu \right)=T\sin c^{2}\left(\pi T\nu \right)\;\;\;when\left(\phi_{0}=\pi \right)$$

Consequently, when driven at the half-wave voltage, the EOM undergoing PRBS modulation generates a broad optical spectrum devoid of carrier and harmonic components, whose spectral width is inverse to the bit period of the PRBS. The spectral of a laser subject to phase modulation $S_{E}\left(\nu \right)$ is the convolution of the intrinsic laser spectrum $S_{0}\left(\nu \right)$ and spectrum variations induced by phase modulation $S_{PRBS}\left(\nu \right)$ [39].

(6) $$S_{E}\left(\nu \right)=S_{0}\left(\nu \right)*S_{PRBS}\left(\nu \right)$$

where ∗ represents convolution. For the phase variations induced by PRBS modulation, $S_{PRBS}\left(\nu \right)$ is centered at zero frequency; thus, the convolved spectrum $S_{E}\left(\nu \right)$ is centered at the laser center frequency $\nu_{0}$. By virtue of the convolution property in Equation (6), we derive:

(7) $${\Delta \nu_{e}}^{2}\approx {\Delta \nu_{0}}^{2}+{\Delta \nu_{PRBS}}^{2}$$

The coherence length of the laser, denoted by $L_{c}$, is the maximum propagation distance over which the light wave maintains a consistent phase relationship. This length is intrinsically linked to the coherence time τ, which corresponds to the duration of continuous emission from an atom in the light source. Furthermore, $L_{c}$ is determined by the time required for a complete wave train to pass a fixed point in space [45]

(8) $$L_{c}=\tau c$$

where *c* is the speed of light. Differentiating both sides of Equation (8) yields the relationship between the coherence length $L_{c}$ and the spectral width $\Delta \nu_{e}$:

(9) $$L_{c}=\frac{c}{\Delta \nu_{e}}\approx \frac{c}{\sqrt{{\Delta \nu_{0}}^{2}+{\Delta \nu_{PRBS}}^{2}}}$$

Equation (9) demonstrates that the coherence length $L_{c}$ of the light is inversely proportional to its spectral width $\Delta \nu_{e}$. The light sources used in the IFOHs are narrow-linewidth lasers with kHz-level $\Delta \nu_{0}$ [22]. However, through high-speed EOM modulation, GHz-level spectral broadening is achievable. The spectral broadening effect is illustrated in Figure 1B. As shown Figure 1C, when continuous-wave (CW) light is modulated by a PRBS with an amplitude of ±π/2 (the phase jumps to π), the continuous phase evolution of the light field is disrupted, the coherence of the light decreases. Crucially, the wavelength stability of the modulated light field remains governed by the input CW laser. Thus, the high wavelength stability inherent to the original narrow-linewidth laser is preserved, enabling PRBS phase modulation to precisely control coherence length while maintaining spectral purity.

Based on a parasitic interference mechanism, suppressing the autocorrelation of interrogation light pulses can effectively mitigate parasitic interference noise [5]. Consequently, PRBS phase modulation reduces the coherence length of the laser, thereby achieving superior suppression of parasitic interference noise. Notably, suppression efficacy scales inversely with $L_{c}$.

### 2.2. Impact of Coherence Length on IFOH System Background Noise

Next, we model the relationship between laser coherence length and background noise by analyzing PRBS-induced spectral broadening in a parasitic interference-free IFOH system. In the PGC-modulated IFOH system depicted in Figure 1A, the reference arm of the interferometer (interrogation pulse 1, reflects by FRM0) propagates backward to the left side of FRM0. Similarly, the signal arm (interrogation pulse 0, transmits through the sensing optical fiber and reflects by FRM1) also propagates back to the left side of FRM0. In this configuration, the link fiber length is assumed to be zero and thus contains no RS points and other parasitic interference [5]. The two reflected pulses arrive simultaneously at the left side of FRM0 and interfere while propagating in the same direction. With unit interrogation light amplitude and negligible transmission losses, the optical fields are:

(10) $$\left\{\begin{array}{l} E_{0}=e^{i\left(2\pi \nu_{e}t+\varphi_{l}+2\varphi_{s}+\varphi_{0}+C\cos\left(\omega_{c}t\right)\right)}\\ E_{1}=e^{i\left(2\pi \nu_{e}t+\varphi_{l}+\varphi_{1}\right)} \end{array}\right.$$

where $\nu_{e}$ is the spectrum of the optical pulse after phase modulation by PRBS; $\varphi_{l}$ denotes the propagation phase shift in the transmission optical fiber; $\varphi_{s}$ represents the phase shift induced by the sensing optical fiber carrying the target information; $\varphi_{0}$ and $\varphi_{1}$ denote the initial phases of the reference light and the signal light, respectively; and $C\cos\left(\omega_{c}t\right)$ is the PGC signal introduced into the interrogating optical pulse 0, where $\omega_{c}$ is the PGC angular frequency, and *C* is the modulation depth. Accordingly, the photodetector thus records interference intensity:

(11) $$\begin{array}{cc} I & =\left(E_{0}+E_{1}\right)\cdot {\left(E_{0}+E_{1}\right)}^{*}\\ & =2+2V\cos\left(C\cos\left(\omega_{c}t\right)+2\varphi_{s}+\varphi_{n}\right) \end{array}$$

V represents the interference degree, which is mainly determined by the polarization states of the two interfering light fields; $\varphi_{n}$ represents the initial phase difference in the interfering light, with $\varphi_{n}=\varphi_{1}-\varphi_{0}$. The phase $\varphi_{s}$ can be extracted from Equation (11) using appropriate demodulation algorithms for the received signal [34]. The impact of the reduced coherence length on system noise is reflected in how spectral broadening affects $\varphi_{s}$. When external acoustic vibration perturbs the sensing optical fiber segment wound on the sensing element, the changes in $\varphi_{s}$ for single-mode silica fiber can be approximated as:

(12) $$\varphi_{s}=\frac{2\pi n\Delta L\nu_{0}}{c}\kappa \varepsilon_{z}\cdot \Delta \nu_{e}$$

$\varepsilon_{z}$ represents the longitudinal strain that occurs in the sensing fiber when subjected to longitudinal stress. ΔL represents the length of the sensing fiber. κ is a fixed coefficient related to the sensitization structure of the IFOH. Spectral comparison demonstrates that PRBS phase modulation generates additive phase noise components:

(13) $$\varphi_{\mathrm{noise}}\approx \frac{2\pi n\varepsilon_{z}\nu_{0}}{c}\Delta \nu_{PRBS}2\kappa \Delta L$$

Accordingly, when there is no parasitic interference noise in IFOHs, PRBS phase modulation induced spectral broadening noise, resulting in increased phase noise. Combining Equations (9) and (13), we obtain:

(14) $$\varphi_{\mathrm{noise}}\approx \frac{2\pi n\varepsilon_{z}\nu_{0}}{L_{c}}2\kappa \Delta L$$

According to Equation (14), the increase in phase noise is inversely proportional to the laser coherence length.

Therefore, as coherence length decreases, its capacity to suppress parasitic interference noise strengthens while the induced phase noise concomitantly increases. Consequently, when reducing IFOH background noise through coherence length control, both effects must be balanced.

In summary, as the link length increases, the level of parasitic interference noise significantly rises. To optimize the detection performance (minimize the background noise), the coherent length should be progressively reduced. However, due to the increase in phase noise caused by spectral broadening, there exists a dynamic lower limit that can be achieved.

### 2.3. Simulation of Introduced Phase Noise

Subsequently, we analyze the phase noise due to the coherence length decrease during PGC modulation through numerical simulation. The simulation parameters are listed in Table 1. The laser central wavelength is set to 1550 nm, with an intrinsic linewidth of 2.2 kHz. The PGC modulation signal is characterized by an amplitude of 2.37 and a carrier frequency $f_{PGC}$ of 15.625 kHz. The optical pulse switching frequency occurs at 250 kHz, and the fiber refractive index *n* is 1.47. The time delay of the matched interferometer $\tau_{c}$ is set to 500 ns, which determines the length of the sensing fiber:

(15) $$\Delta L=c\cdot \tau_{c}/2/n$$

The speed of light $c=3\;\times \;10^{8}$ m/s, according to Equation (15), ΔL=51.02 m. The widths of the spectral broadening $\Delta \nu_{PRBS}$ are set to 250, 500 and 1000 MHz, and the corresponding coherence lengths $L_{c}$ are 1.2, 0.6 and 0.3 m, respectively.

**Figure 2:**
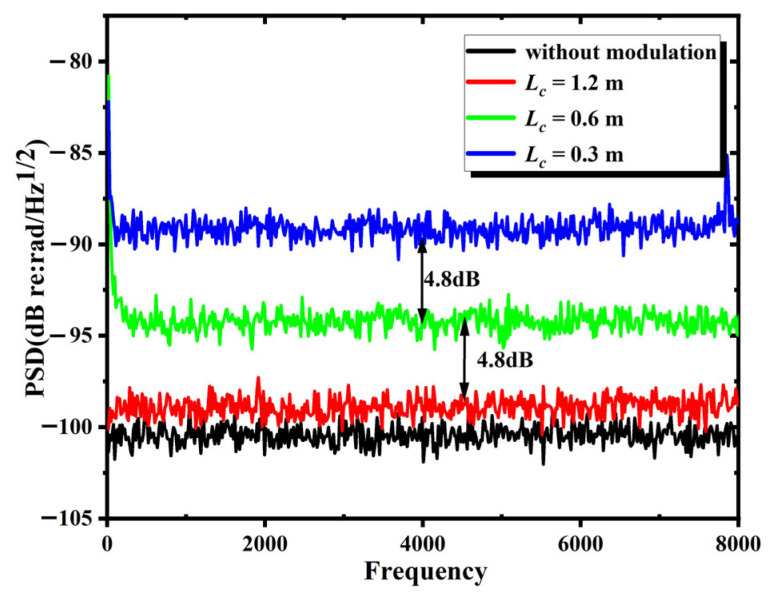
Background noise of IFOH when the coherence length is 1.2 m, 0.6 m or 0.3 m and without modulation.

**Figure 3:**
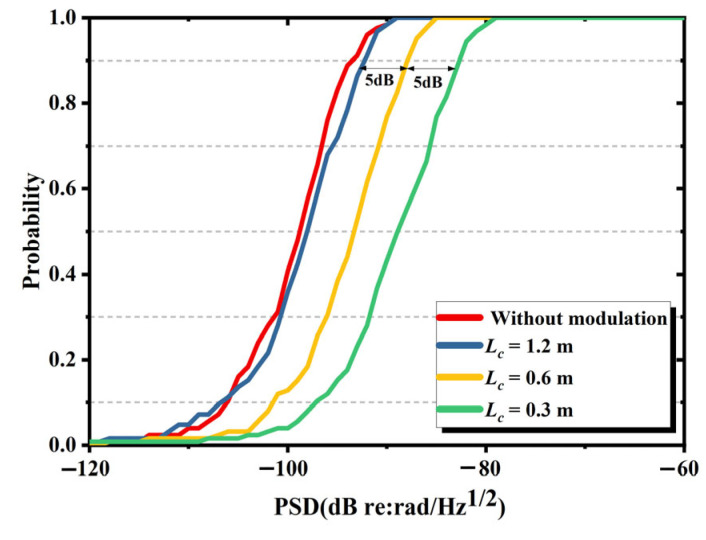
Cumulative probability distribution of the background noise at 1 kHz when the coherence length is 1.2 m, 0.6 m or 0.3 m and without modulation.

A total of 125 independent simulation experiments were carried out under different coherence length conditions. Experimental data were averaged across replicate trails, with the results presented in Figure 2. Figure 3 illustrates the cumulative probability distributions of noise at 1 kHz under varying coherence lengths. Based on engineering standards, the system background noise level is defined as the 90% probability noise. Both averaged noise analysis and noise distribution-based characterization reveal three statistical characteristics: (i) decreasing coherence length led to a rise in the IFOH background noise in the absence of parasitic interference; (ii) as the coherence length increased sufficiently, the IFOH phase noise approached the level observed without modulation, at which point other factors determined the background noise; and (iii) within a specific range, the background noise of IFOH increases by 5 dB ($\mathrm{re}:\mathrm{rad}/\sqrt{\mathrm{Hz}}@1\mathrm{kHz}$) when the laser coherence length is halved. Based on the calculation formula of PSD

(16) $$\mathrm{PSD}=20\mathrm{lg}\left(\varphi_{\mathrm{noise}}/f_{s}/N\right)$$

and Equation (14), halving $L_{c}$ should theoretically result in an approximate 6 dB decrease in the background noise PSD, which is roughly consistent with the simulation results.

## 3. Experimental Results and Discussion

### 3.1. System Configuration

The experimental setup employs a laser source (RIO Inc., Santa Clara, CA, USA) with a central wavelength of 1549.96 nm, exhibiting intensity noise and phase noise levels of −120√HzdB @ 1 kHz and −110 dB√Hz @ 1 kHz, respectively. Continuous-wave output from this laser is modulated into 480 ns optical pulses at 250 kHz through the AOM (G&H Inc., Ilminster, UK), achieving an extinction ratio exceeding 50 dB. Optical signals then propagate through a self-built polarization-maintaining fiber interferometer comprising two key elements: CIF with 102 m arm imbalance (500 ns delay) and a piezoelectric transducer (PZT) mounted on its short arm for PGC modulation. The PGC was operated at 15.625 kHz with modulation amplitude C = 2.37. The link fiber utilizes standard G652D fiber, while the optical circulator and 5% coupler (Fiber Home Communications Inc., Wuhan, China) integrate the FRMs (Lightpromotech Inc., Beijing, China) with a reflectivity over 99%. A manually adjustable optical attenuator is placed before the PD to fine-tune the intensity of the laser pulse received by the PD, ensuring that the collected electrical signal is within an appropriate range. The NI-6131 acquisition card produced by NI is used for generating the modulation signal and sampling the received signal. The demodulation software processed interference pulses with 16,384 samples per iteration.

**Figure 4:**
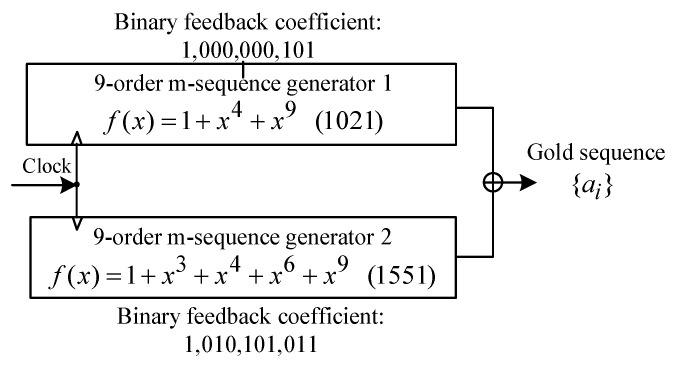
The generation method and timing of PRBS.

For PRBS phase modulation, an Arbitrary Waveform Generator (AWG, SPECTRUM M4i.6631, Grosshansdorf Germany ) generates a 9-order Gold sequence (length: 2^9^ − 1 = 511) based on a modulo-2 sum structure, shown in Figure 4. The AWG output is amplified by a DR-AN-10-MO RF amplifier (iXblue Inc., Saint-Germain-en-Laye, France) and drives an EOM (iXblue Inc.)to apply the phase modulation.

### 3.2. The Influence of Coherence Length on Interference Visibility

**Figure 5:**
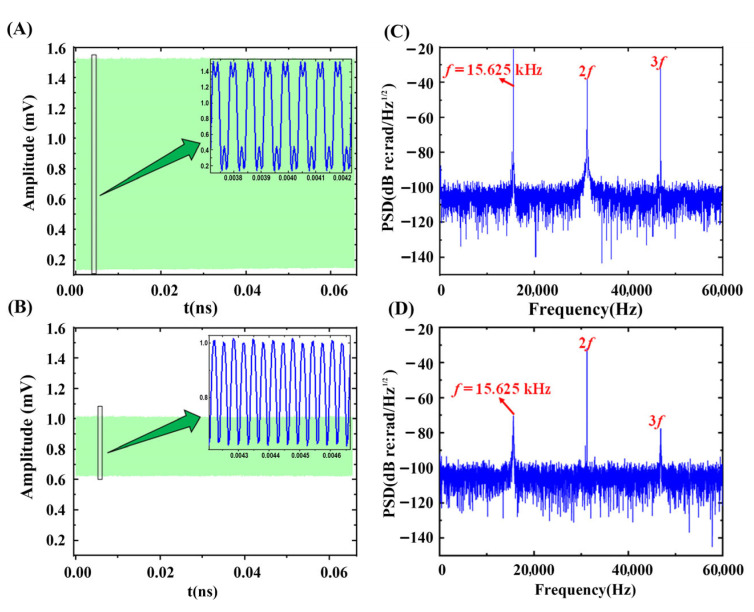
Interference signal with and without coherence control. (**A**) Data points from interfering pulses without coherence control. (**B**) Data points from interfering pulses with coherence control. (**C**) The frequency-domain graph of the interference signal without coherence control. (**D**) The frequency-domain graph of the interference signal with coherence control.

As shown in Figure 5, when the link fiber length is zero (no parasitic interference is present), the introduction of coherence length control into the IFOH system resulted in a reduction in the visibility of the interference signal. In the frequency domain, this manifested as a decrease in the PSD amplitude of the PGC modulation frequency and its harmonics, accompanied by an increase in the background noise at all frequencies.

**Figure 6:**
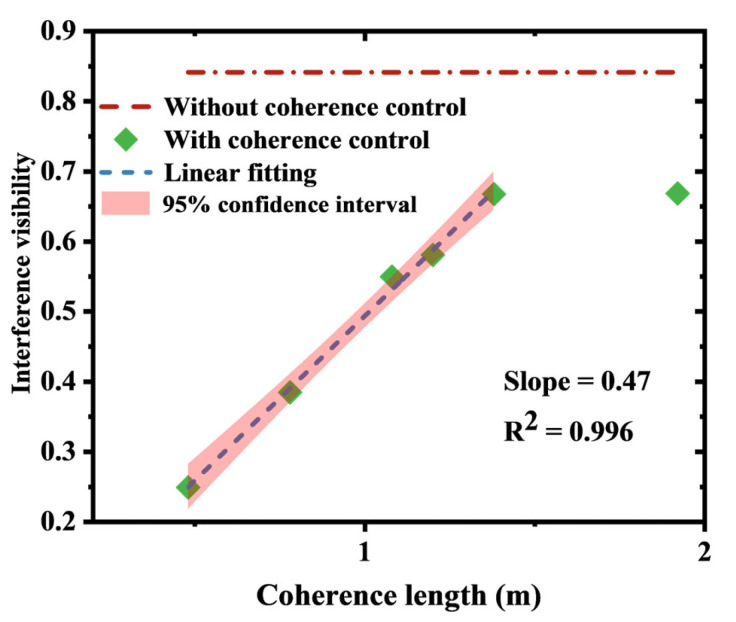
The relationship between the bit periods of different coherence length and the interference visibility.

We can calculate the interference visibilities across varying coherence lengths. As shown in Figure 6, for visibilities below 0.65 (corresponding to coherence lengths below 1.38 m), the cross-correlation coefficient between coherence length and visibility is 0.996. This indicates a strong linear relationship within this range (coherence length from 0 to 1.38 m), with a slope of 0.47. However, when the coherence length reaches 1.92 m, the interference visibility operates outside the linear range. The underlying physical mechanism is defined by three distinct phases: near-zero coherence length yields negligible interference with visibilities approaching zero; increasing coherence length initiates a linear response regime where visibility scales proportionally with coherence length; and once coherence length exceeds the threshold for sustained interference (≈1.38 m), visibility growth saturates and transitions into a nonlinear phase, thereby explaining the divergence from the fit observed at 1.92 m.

### 3.3. The Influence of Coherence Length on Background Noise of IFOH Without Parasitic Interference

**Figure 7:**
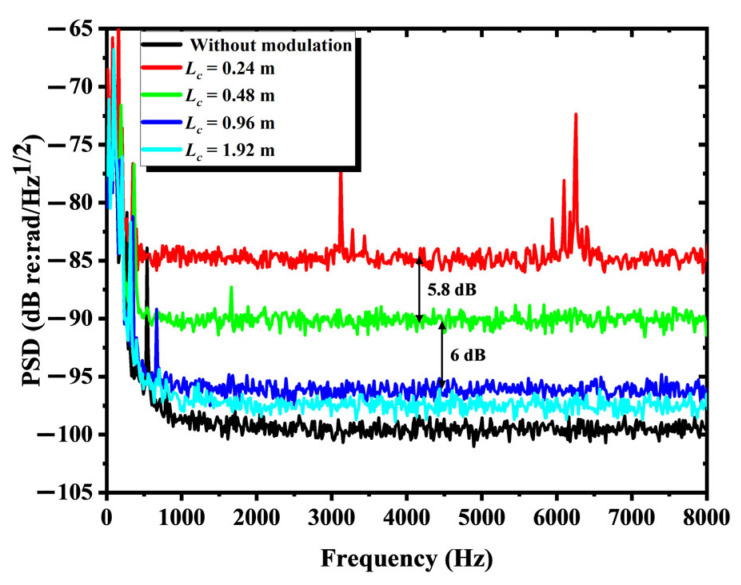
Background noise of IFOH when the coherence length is 0.24 m, 0.48 m, 0.96 m or 1.92 m and without modulation.

**Figure 8:**
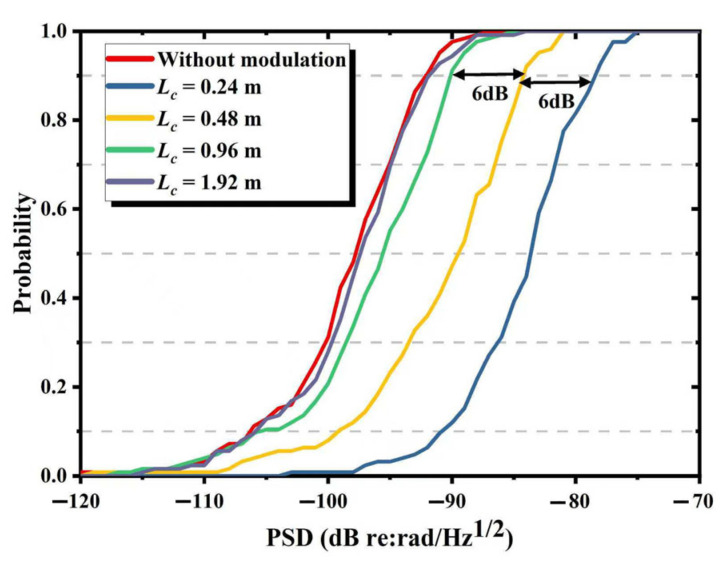
The probability distribution of the background noise when the coherence length is 0.24 m, 0.48 m, 0.96 m or 1.92 m and without modulation.

To investigate the quantitative relationship between the coherence length and the phase noise introduced by the reduction in interference degree, we analyzed the background noise of the IFOH under different coherence lengths when the link fiber was 0. As shown in Figure 7, when the coherence lengths are 0.24 m, 0.48 m, 0.96 m and 1.92 m, respectively, the average PSD of the background noise for 125 sets of test data is −84.2 dB (re: $\mathrm{rad}/\sqrt{\mathrm{Hz}}@$ 1 kHz), −90.0 dB, −96.0 dB and −98.6 dB, while the PSD of the background noise without modulation is −99.2 dB. Meanwhile, as shown in Figure 8, the 90% cumulative probability noise values corresponding to these five groups of coherence lengths are −78 dB, −84 dB, −90 dB, −92 dB and −92 dB, respectively. It can be found that, when the coherence length gradually reduced from 0.96 m to 0.48 m and 0.24 m, the average noise increased by 5.8 dB and 6 dB, respectively, and the 90% cumulative probability noise values all showed a rise of 6 dB. This result is highly consistent with the model obtained in Section 2.

It is worth noting that when the coherence length is reduced from 1.92 m to 0.96 m, the increase in the system’s background noise is significantly less than 6 dB. By analyzing the relationship between the coherence length and the interference visibility within this range (as shown in Figure 6), it is found that they have deviated from the linear relationship region. This result further confirms that the increase in background noise due to coherence length reduction is primarily caused by decreased interference visibility.

Therefore, when suppressing parasitic interference noise by the coherence length control, it is essential to take into account the increase in the background noise caused by the decline in the interference visibility. For different intensities of parasitic interference (primarily RS caused by long-link optical fiber) and the different time delays of unequal-arm interferometers, the optimal system coherence length needs to be determined through experiments.

### 3.4. The Optimal Coherence Length Under Different Fiber Link Lengths

**Figure 9:**
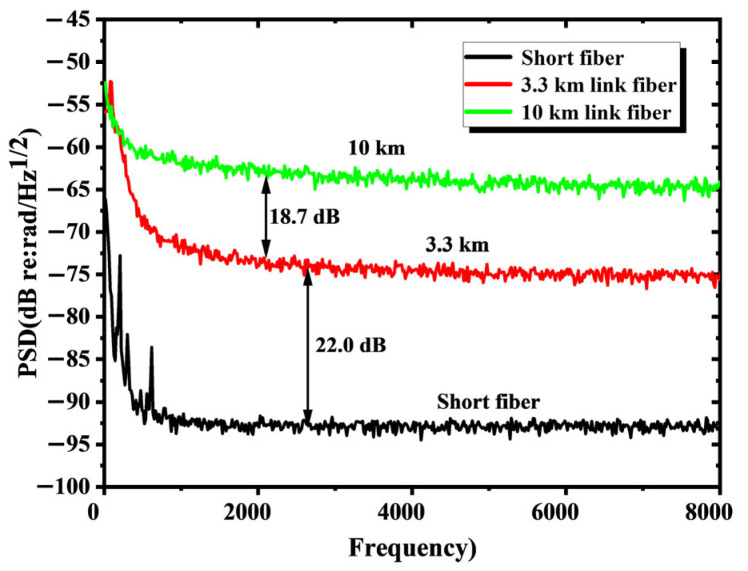
The background noise of IFOH under optical fibers with varying link lengths.

In IFOH, the RS intensities of link fibers with different lengths are distinct. The longer the link fiber is, the stronger the parasitic scattering intensity becomes, as depicted in Figure 9.

**Figure 10:**
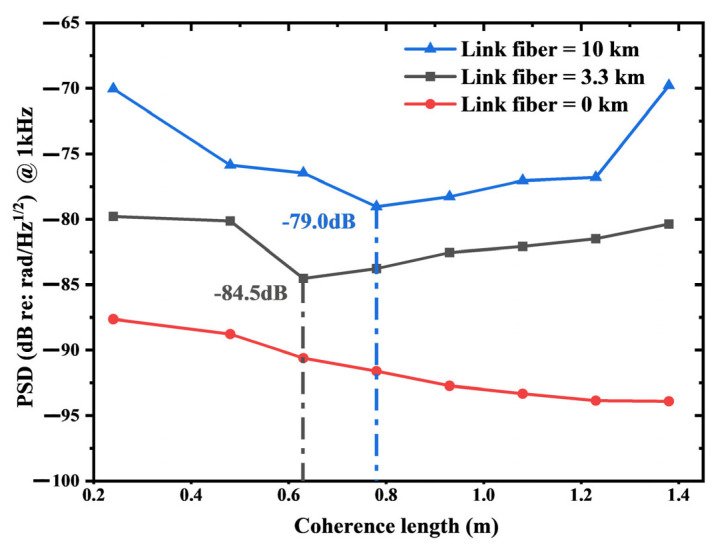
The relationship between the coherence length and the background noise when the link length is 0, 3.3 or 10 km.

Longer link fibers necessitate shorter coherence lengths, yet excessively short coherence lengths result in insufficient interference visibility, thereby raising background noise. Consequently, to ascertain the optimal coherence lengths for link fibers of a certain length, taking 0, 3.3 and 10 km as instances, we conducted experiments and present the results in Figure 10. The experimental results demonstrate that the background noise of the IFOH system exhibits distinct variations with respect to coherence length under different link lengths. In the absence of the link fiber, the background noise decreases monotonically as the coherence length increases, ranging from −87.6 dB at 0.24 m to −94.0 dB at 1.38 m. However, upon introducing the link fiber into the system, a significant change in the noise behavior is observed. For the 3.3 km link fiber, the noise initially decreases to a minimum of −84.5 dB at 0.93 m and subsequently rises to −80.4 dB at 1.38 m. Similarly, for the 10 km link fiber, the noise reaches its lowest value of −79.0 dB at 0.78 m before the presence of parasitic interference, the relationship between background noise and coherence length follows a nonlinear pattern—first decreasing and then increasing. Moreover, the optimal coherence length diminishes with increasing link fiber length; specifically, it is 0.93 m for the 3.3 km link and reduces to 0.78 m for the 10 km link.

These results align with our theoretical predictions. The reason why the background noise first decreases and then rebounds with the increase in the coherence length is that an excessively low coherence length will lead to a reduction in the interference visibility, thereby elevating the background noise. Meanwhile, a larger coherence length is insufficient to adequately suppress the stronger parasitic interference noise. Therefore, as the link length of the IFOH system increases, the optimal coherence length of the corresponding laser gradually decreases.

Notably, by determining the optimal coherence length of the laser, this study achieves a further reduction of 4.5 dB in the background noise level of the IFOH system with a 3.3 km link fiber without changing the experimental structure [5].

## 4. Discussion

The experimental performance of digitally generated white noise is comparable to that of PRBS modulation [46]. This equivalence stems from the fundamental nature of digitally synthesized “white noise” as pseudo-random sequences generated by deterministic algorithms. The practical application of white noise in real-world environments is constrained by limitations in data acquisition and output hardware, making it difficult to implement in practice. Furthermore, our analysis reveals that white noise corresponds to an extremely short “bit period”, which translates to a very low coherence length. As previously noted, excessively low coherence lengths—due to reduced interference visibility—can actually lead to increased noise levels. Therefore, the PRBS scheme with controllable coherence length represents the most viable and optimal approach under current technical constraints.

To validate the universality of the theoretical model, we modified both the light source’s central wavelength (1533 nm, 1540 nm, 1550 nm) and the arm-length difference in the unbalanced CIF. The experimental results demonstrate that the background noise level remains consistent across different wavelengths, confirming the wavelength independence of background noise. Furthermore, as discussed in Section 2.1, the intrinsic linewidth of narrow-linewidth lasers is typically kHz-level, whereas PRBS modulation broadens the spectrum to GHz-level. Consequently, this conclusion applies universally to all interferometric systems employing narrow-linewidth lasers. Additionally, when the time delay of the unbalanced CIF was adjusted to 244 ns, the optimal coherence length under varying link lengths diverged from that of the 500 ns system. Nevertheless, its monotonically decreasing trend with increasing link length aligns with theoretical predictions, robustly demonstrating the strong universality of our theoretical framework across time-delay variations. Critically, when the time delay of the IFOH is 500 ns, for a 3.3 km link, an optimal coherence length of 0.93 m reduces the background noise level of −84.5 dB @ 1 kHz. This represents an additional 4.5 dB of suppression compared to the −80 dB baseline achieved in prior studies.

## 5. Conclusions

This study systematically explores the dual-effect mechanism of PRBS phase modulation on IFOH performance through integrated theoretical modeling and experimental validation. Building on this principle, we establish the optimal coherence length for various link lengths. By determining the optimal coherence length, the system noise level can be further suppressed, achieving an additional reduction of 4.5 dB. This technological advancement significantly improves the sensitivity in detecting weak signals of the IFOH, such as those from marine organisms and ship radiated noise, thereby improving measurement precision and data reliability. Furthermore, the proposed optimal coherence length selection strategy provides a more reliable data foundation for long-link IFOH research and facilitates high-precision marine scientific investigations.

## Figures and Tables

**Table 1:** Simulation parameters of the signal.

$\Delta \nu_{\mathrm{las}}$	*C*	*f_PGC_*	*f_AOM_*	*L_c_*	$\varepsilon_{z}$
2.2 kHz	2.37	15.625 kHz	250 kHz	1.2, 0.6, 0.3 m	0.01

## Data Availability

The data presented in this study are available on request from the corresponding author.
